# Ephemeral signatures of phylosymbiosis in dermal microbiomes within the requiem shark family (Carcharhinidae)

**DOI:** 10.1099/acmi.0.001140.v3

**Published:** 2026-03-19

**Authors:** Ifan Dafydd Lynn, Marius Alexander Wenzel

**Affiliations:** 1 School of Biological Sciences, University of Aberdeen, Tillydrone Avenue, AB24 2TZ, UK

**Keywords:** Carcharhinidae, co-evolution, dermal, metagenetics, microbiome, phylosymbiosis

## Abstract

The elasmobranch dermal microbiome may be important for buffering effects of environmental stress on host health and population viability via functional metabolic interactions. Dermal microbiomes among elasmobranch orders co-vary with host phylogeny (phylosymbiosis), indicating functional co-evolution with their hosts at deep phylogenetic splits. However, the extent of phylosymbiosis and potential for functional co-evolution within particularly species-rich elasmobranch families remains unknown. Here, we re-analyse Illumina amplicon sequence data from the 16S rRNA gene from eight Carcharhinid shark species (plus one Ginglymostomatid outgroup) across six independent studies and explicitly examine the extent of phylosymbiosis in dermal microbiomes within this family. We found extensive divergence in operational taxonomic unit (OTU) abundance and functional metabolic capacity between studies, driven by disparity in OTU sharing and probably reflecting geographical and seasonal factors. Total microbiome structure was incongruent with shark phylogeny, providing no evidence for phylosymbiosis when considering all species and OTUs. However, using bootstrapping and subsampling methods, we identified several subsets of OTUs where Bray–Curtis dissimilarity supported perfect topological congruence with shark phylogeny or strong associations with phylogenetic distances, but not both. Partial Mantel tests identified ten candidate OTUs that supported a moderately strong signal of phylosymbiosis across all shark species and included the immunostimulant skin symbiont genera *Lactiplantibacillus* and *Alcaligenes*. Overall, this provides provisional evidence for phylosymbiosis in a minority fraction of the elasmobranch dermal microbiome within the Carcharhinidae family and will necessitate coordinated large-scale studies to establish the generality of these findings.

## Data Summary

The authors confirm that all supporting data, code and protocols have been provided within the article or through supplementary data files. Code is available from https://github.com/wenzelm/lynn.

## Introduction

Phylosymbiosis is an eco-evolutionary phenomenon that is evident as congruence between phylogenetic relationships of host organisms and compositional similarity of their microbiomes [1]. Identifying such evolutionary host-microbe associations is an important step towards characterizing the ecological and physiological function of microbial symbionts [2,3]. This may then lead to improved understanding of functional interactions within the holobiont, which is particularly critical for ascertaining environmental risk factors for microbial disruption (dysbiosis) such as pollution or climate change that may impact population viability [4]. Although phylosymbiosis is frequently reported for diverse groups of organisms across deep phylogenetic splits [2,3] and even at the genus level [5,7], our lack of understanding of its prevalence and mechanisms at each level of phylogenetic divergence hinders its utility in applied biological conservation [8,9].

Elasmobranch fishes (sharks, rays and skates) are particularly powerful study systems for phylosymbiosis both on a fundamental level and for application to acute conservation issues in oceanic ecosystems. Elasmobranchs are globally important marine keystone organisms since they are high-level mesopredators that control lower trophic levels, prevent overgrazing and aid in nutrient cycling of reef environments [10,13]. Rapid changes in the oceanic environment have caused 90–92% declines in some batoid and selachimorph populations [14,16], which may be associated with dysbiosis of the microbiome and consequential negative physiological implications for nutrition and health [17,18]. The shark dermal microbiome is implicated in preventing skin infections by producing antibiotic compounds [19,20] and by metabolizing heavy metals such as cobalt, zinc and cadmium to counteract bioaccumulation [21,22]. The unique denticle morphology of shark skin prevents biofilms from forming [23,24] and thus recruits microbial communities that are distinct from those of teleost fishes [25]. Shark dermal microbiomes are highly diverse [18], distinct from the environmental microbiome [26,29] and other anatomical locations [26,28,30, 31], and may be subject to vertical inheritance [32], providing an intriguing case study system for examining the causes and consequences of phylosymbiosis.

Several studies have reported phylosymbiosis of dermal microbiomes across elasmobranch orders, though each study used only a few species from two to three orders [25,31, 33]. These studies suggest a possible functional host–microbiome relationship at the elasmobranch order level, but the extent of such a relationship at shallower phylogenetic divergence is unknown. Here, we examine phylosymbiosis within species of the Carcharhinidae (requiem sharks) family. Carcharhinids are a particularly diverse and shallowly diverged elasmobranch radiation with controversial phylogeny [34,36] that was only recently resolved by extensive multi-locus DNA sequencing data [37]. While several studies have characterized carcharhinid dermal microbiomes, highlighting structure among individuals [28], geographical locations [20,27], years [27,28] and species [26,29], the extent of phylosymbiosis within this family has not been explicitly examined. Here, we combine samples from multiple studies to maximize species representation and examine the extent to which geographical and temporal environmental variation may impact the identification of phylosymbiosis at shallow phylogenetic divergence.

## Methods

### Sequence data acquisition and processing

Illumina 16S rRNA amplicon sequence data of Carcharhinidae dermal samples were obtained from the NCBI Sequence Read Archive (SRA) or the European Nucleotide Archive (ENA) (Table S1, available in the online Supplementary Material). A total of 17 samples across nine species from six independent studies were included [20,26,29, 31], comprising Caribbean reef shark (*Carcharhinus perezii*), dusky shark (*Carcharhinus obscurus*), sandbar shark (*Carcharhinus plumbeus*), blue shark (*Prionace glauca*), blacktip reef shark (*Carcharhinus melanopterus*), lemon shark (*Negaprion brevirostris*), Atlantic sharpnose shark (*Rhizoprionodon terraenovae*), tiger shark (*Galeocerdo cuvier*) and the outgroup nurse shark (*Ginglymostoma cirratum*). Two species were available from two studies of different global regions (blacktip reef shark: West Atlantic vs. Seychelles; sandbar shark: West Atlantic vs Eastern Mediterranean) [20,26, 28], three species were available from multiple years at the same (dusky shark and sandbar shark [28]) or similar (tiger shark [27]) locations and one study (bioproject PRJNA393438) comprised six species from the same location [26] (hereafter: ‘core samples’). Additionally, five control seawater samples across four studies [26,29] were included to enable identification of geographical and temporal environmental effects on microbiome composition. All studies used skin swabs but varied in anatomical location and PCR primer choice (V4, V5-V6 and V6-V8; Table S1).

Sequence data were trimmed, filtered and assembled to amplicon sequence variants (ASVs) in DADA2 1.36 [38], trimming primers where required. Data from each study were processed independently with tuned parameters for truncation and filtering (Table S2). ASVs were inferred in pooled-sample mode, and bimeric ASVs were removed using the consensus method. Multiple libraries of the same species, location and year within the same study were merged where required to maximize read depth (Table S2). ASVs were taxonomically annotated with the RDP Naive Bayesian Classifier algorithm [39] against the silva nr99 V138.2 database. To enable comparative analysis across studies that used different primer pairs, ASVs with the same genus annotation were agglomerated into operational taxonomic units (OTUs) (e.g. 40,41). For corroboration, alternative datasets were generated by agglomerating by family, order and class ranks. Singleton OTUs (<2 total counts) and contaminant OTUs (annotated as archaea, chloroplast or mitochondria) were removed. OTUs were functionally annotated using the FAPROTAX 1.2.11 database [42].

### Microbiome structure analysis

OTU counts and taxonomic annotations were analysed in R 4.5.0 using the *phyloseq* package [43]. Rarefaction curves were generated for each sample using *ggrare* from the *ranacapa* package [44], and data were rarefied to the lowest observed read depth across all samples to account for two orders of magnitude range in sequencing effort across studies. Shannon and inverse Simpson alpha diversity metrics were obtained for each sample and compared between core and non-core samples using Wilcoxon rank-sum tests. Jaccard distances or Bray–Curtis distances among samples were hierarchically clustered into dendrograms using Ward’s method and tested for association with geographical location or study using distance-based redundancy analysis and permutational multivariate analysis of variance (PERMANOVA) with 9999 permutations (*vegan* package [45]). Statistical support values for each branch in the dendrograms were obtained by bootstrapping across OTUs 1,000 times and calculating the proportion of replicates containing the branch (*ape* package [46]).

Phylosymbiosis was first examined by comparing a cladogram of host phylogenetic relationships to dendrograms of hierarchically clustered Bray–Curtis microbiome dissimilarities (Ward’s method) using clustering information distance (CID) [47] as implemented in the *TreeDist* package [48]. Statistical significance of CID was obtained by shuffling tip labels across the microbiome dendrogram 1,000 times and calculating the proportion of replicates with lower CID than the point estimate [8]. Second, associations between Bray–Curtis microbiome dissimilarity and host phylogenetic distance (Tamura–Nei+gamma nucleotide distance across 16 genes [37]) were identified using Mantel tests with 1,000 permutations. The nurse shark outgroup was removed from Mantel tests because nucleotide sequence data was not available [37]. Distributions of CID and Mantel correlation coefficients across OTU sets were examined by bootstrapping across OTUs or by subsampling ten OTUs without replacement 10,000 times.

Since species were unevenly distributed across the six studies (including several singleton species), all analyses of microbiome structure and phylosymbiosis were first carried out on the six core samples from the same study (bioproject PRJNA393438) before attempting to generalize results to the full dataset, following recommended practice for confounded meta-studies when direct modelling batch effects is not possible [49]. Partial Mantel tests were used to account for variation in within-study and between-study species comparisons in phylosymbiosis tests by conditioning for between-study variation with a binary distance matrix (0=same study; 1=different studies). To characterize functional study-specific effects, differences in abundances of individual OTUs or functional terms between the core samples and all other samples were identified using *DESeq2* 1.48 [50].

## Results

### Extensive spatio-temporal disparity of Carcharhinid dermal microbiomes

**Fig. 1.**
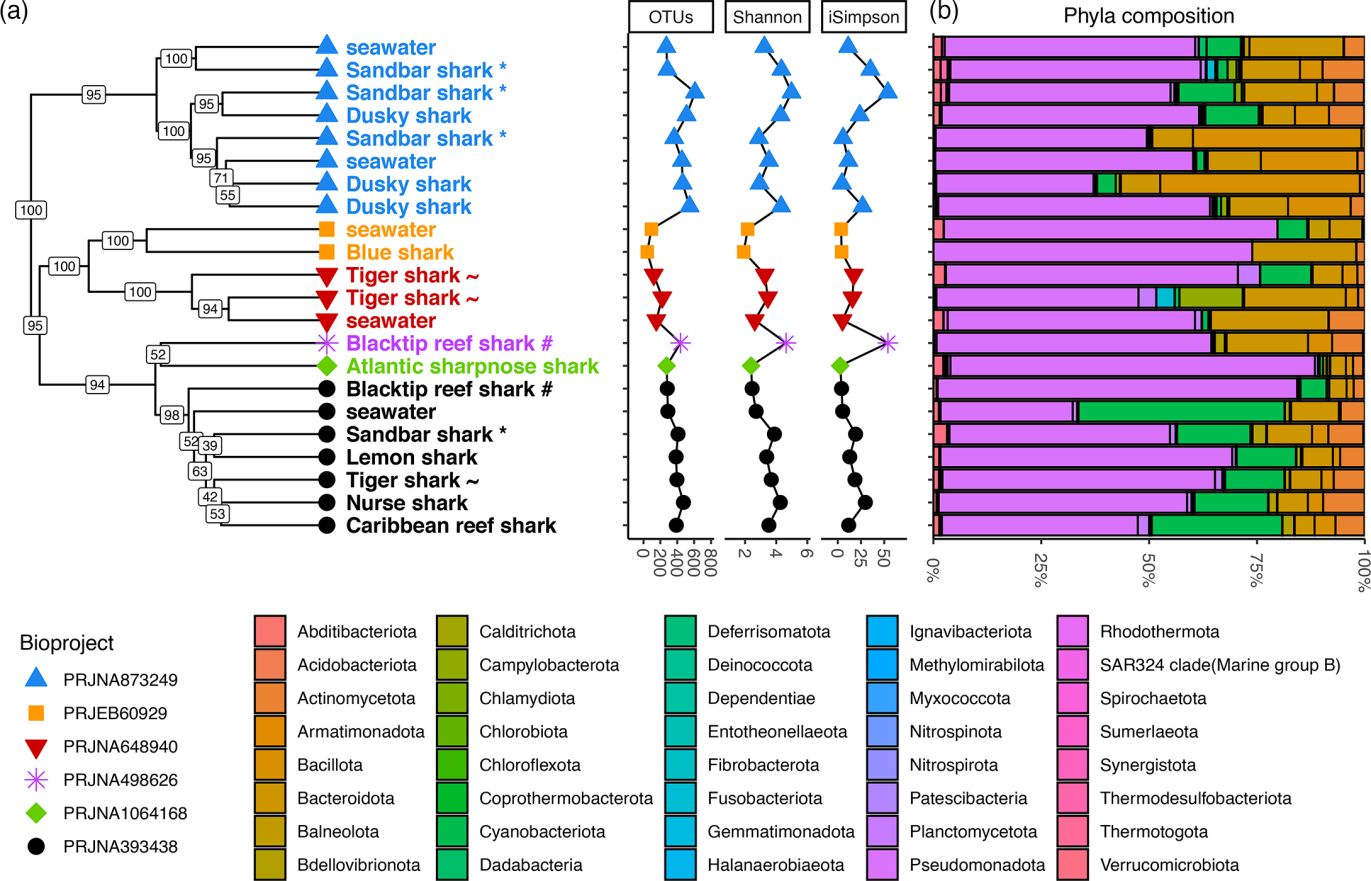
Summary of microbiome composition among shark dermal microbiomes and environmental seawater samples (1,328 OTUs). (**a**) Hierarchical clustering dendrogram (Ward’s method) of Jaccard distance among microbial genera (left), next to alpha diversity metrics (number of OTUs, Shannon index and inverse Simpson index). Tip symbols and colours indicate six independent studies (bioprojects; Table S1). Species replicates across studies are highlighted with special characters (* sandbar shark; ~ tiger shark; # blacktip reef shark). Branch labels indicate support values from 1,000 bootstrap replicates across OTUs. (**b**) Taxonomic composition among OTUs of each sample, agglomerated by phylum (40 phyla). The most frequently observed phyla were *Pseudomonadota*, *Bacteroidota*, *Cyanobacteriota* and *Bacillota* (Table S3).

Sequencing effort ranged from 30,185 to 1,677,653 reads per sample, which were assembled to 22,656 ASVs across all samples (634–10,017 ASVs per study; Table S2). Taxonomic annotation rates were 97.9% (phylum), 95.3% (class), 89.3% (order), 74.5% (family) and 45.4% (genus). The ASVs were agglomerated to 1,465 OTUs with genus annotation (52–778 OTUs per sample; Table S2). After rarefying to 30,185 counts, 45–606 OTUs per sample were retained (1,328 OTUs in total), capturing most of the OTU diversity (Fig. S1). Alpha diversity metrics were variable across samples and studies (Fig. 1a), though there was no significant difference in any metric between the six core samples (bioproject PRJNA393438) and all other samples (Wilcoxon rank-sum test: *W*=50, *P*=0.891).

Jaccard distance among samples indicated significant disparity of OTU sharing among studies (PERMANOVA: *F*_5,16_=3.553, *P*<0.001, *R*^2^=0.526) such that seawater samples clustered correctly with shark samples from the same study, but samples from the same shark species that were collected in multiple studies were dissimilar (Fig. 1a). Only 9 OTUs out of 1,312 (0.7%) were shared among all 17 shark samples, only 15 OTUs were present in at least 1 shark sample in each of the 6 studies, and only 183 OTUs were present in at least 1 shark sample in each of 3 studies that used the same primer pair (PRJNA393438, PRJNA648940 and PRJNA873249; Fig. 1a). This disparity among studies remained when aggregating OTUs taxonomically to family (16 core out of 456 total families), order (13 core out of 198 total orders) or class (6 core out of 78 total classes) ranks, highlighting extensive differences in taxonomic composition of both seawater and shark dermal microbiomes in different geographical or temporal environmental contexts. Notwithstanding, the most frequently observed bacterial phylum (40 total phyla) in all but 1 sample was *Pseudomonadota* (*Proteobacteria*), followed by *Bacteroidota*, *Cyanobacteriota* and *Bacillota* in varying rank order across samples and studies (Fig. 1b, Table S3).

Strong disparity between studies was also apparent in the functional capacity of the microbiomes. Out of all 1,328 OTUs, 35.5% were annotated to 49 functional terms. These terms were significantly disparate among studies based on Jaccard distance (PERMANOVA: *F*_5,16_=4.723, *P*<0.001, *R*^2^=0.596; Fig. S2A). Comparing non-core samples to core samples, 20 terms were significantly differentially abundant (Fig. S2B), such that non-core samples were enriched in terms related to nitrogen, sulphur and chlorate metabolism, xylanolysis, ureolysis, fermentation and photoheterotrophy, and depleted in photoautotrophy and pathogenicity (Fig. S2C).

### Ephemeral phylosymbiosis within fractions of the microbiome

**Fig. 2.**
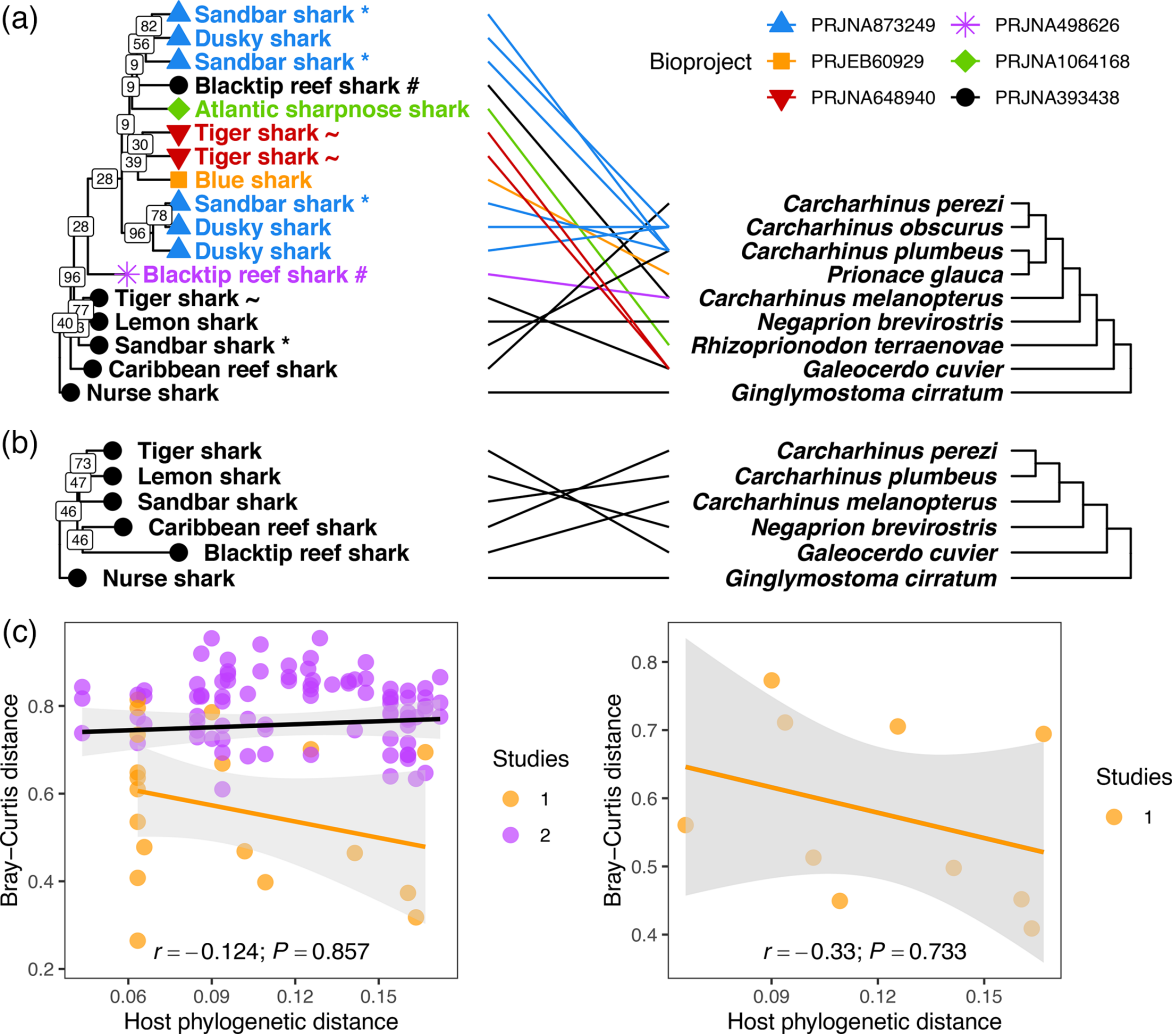
Absence of phylosymbiosis in requiem shark dermal microbiomes. Tanglegrams illustrate the lack of correspondence between microbiome structure (Bray–Curtis dissimilarity; left) and host phylogenetic relationships (right) for 17 samples from 6 independent studies (a; 1,312 OTUs) or 6 core samples from a single study (bioproject PRJNA393438) (b; 787 OTUs). Tip symbols and colours indicate studies (bioprojects; Table S1). Species replicates across studies are highlighted with special characters (* sandbar shark; ~ tiger shark; # blacktip reef shark). Branch labels on dendrograms indicate support values from 1,000 bootstrap replicates across all OTUs. The host cladogram was reproduced from [37], and the dendrograms were rerooted at the nurse shark outgroup (*G. cirratum*). Lines indicate correspondence between species. Both sets of trees are statistically incongruent (CID=0.829; *P*=0.371 and CID=0.749; *P*=0.457, respectively). (**c**) Relationships between Bray–Curtis distance and host phylogenetic distance among all samples (left) or core samples (right), without the nurse shark outgroup. Colours indicate whether each distance was derived from samples within the same study or across two studies. Linear models are fitted (black: all data; yellow: within-study data only) and partial Mantel correlation coefficients (*r*) and *P*-values (1,000 permutations) for the overall relationships (conditioning for across-study comparisons where applicable) are given at the bottom of the panels.

Microbiome structure (Bray–Curtis dissimilarity) among all 17 shark samples from all 6 locations varied significantly by study (PERMANOVA: *F*_5,11_=2.669, *P*<0.001, *R*^2^=0.548), consistent with lack of OTU sharing. Out of 1,312 OTUs, 139 (10.1%) were significantly differentially abundant between core and non-core samples, though 66.7% were untestable due to low average counts (<2). There was no congruence with host phylogeny for all 17 samples (clustering information distance: CID=0.829, *P*=0.371; 1,312 OTUs; Fig. 2a) or the six core samples alone (CID=0.749, *P*=0.457; 787 OTUs; Fig. 2b). In support of topology-based results, partial Mantel tests indicated no significant relationships between Bray–Curtis dissimilarity and phylogenetic distance among all samples (*r*=−0.124, *P*=0.857) or the core samples alone (*r*=−0.330, *P*=0.733; Fig. 2c). Agglomerating OTUs at higher taxonomic ranks (family, order and class) or removing samples from species replicates or outgroup species from different families (nurse shark, tiger shark) had no qualitative effects on these results. Similarly, the functional structure of the dermal microbiomes was also unrelated to host phylogeny (CID=0.810, *P*=0.294; Fig. S3A, B) and uncorrelated with phylogenetic distance (*r*=−0.171, *P*=0.968; Fig. S3C).

Despite this absence of evidence of phylosymbiosis in the whole microbiome, two observations suggested that fractions of the microbiome might display structure consistent with phylosymbiosis. First, the 9 core OTUs among all 17 samples yielded statistically significant, though incomplete, topological similarity (CID=0.752, *P*=0.044; Fig. S4A) and a weak positive distance-based relationship with host phylogeny (*r*=0.161, *P*=0.106; Fig. S4C), although this was not the case for the 142 core OTUs present among the 6 core samples (CID=0.730, *P*=0.364; *r*=−0.247, *P*=0.633; Fig. S4B, C). Second, the low bootstrap support (≤50%) for some branches in the whole-microbiome Bray–Curtis dendrograms indicated a degree of disagreement in microbiome structure among sets of OTUs (Fig. 2).

**Fig. 3.**
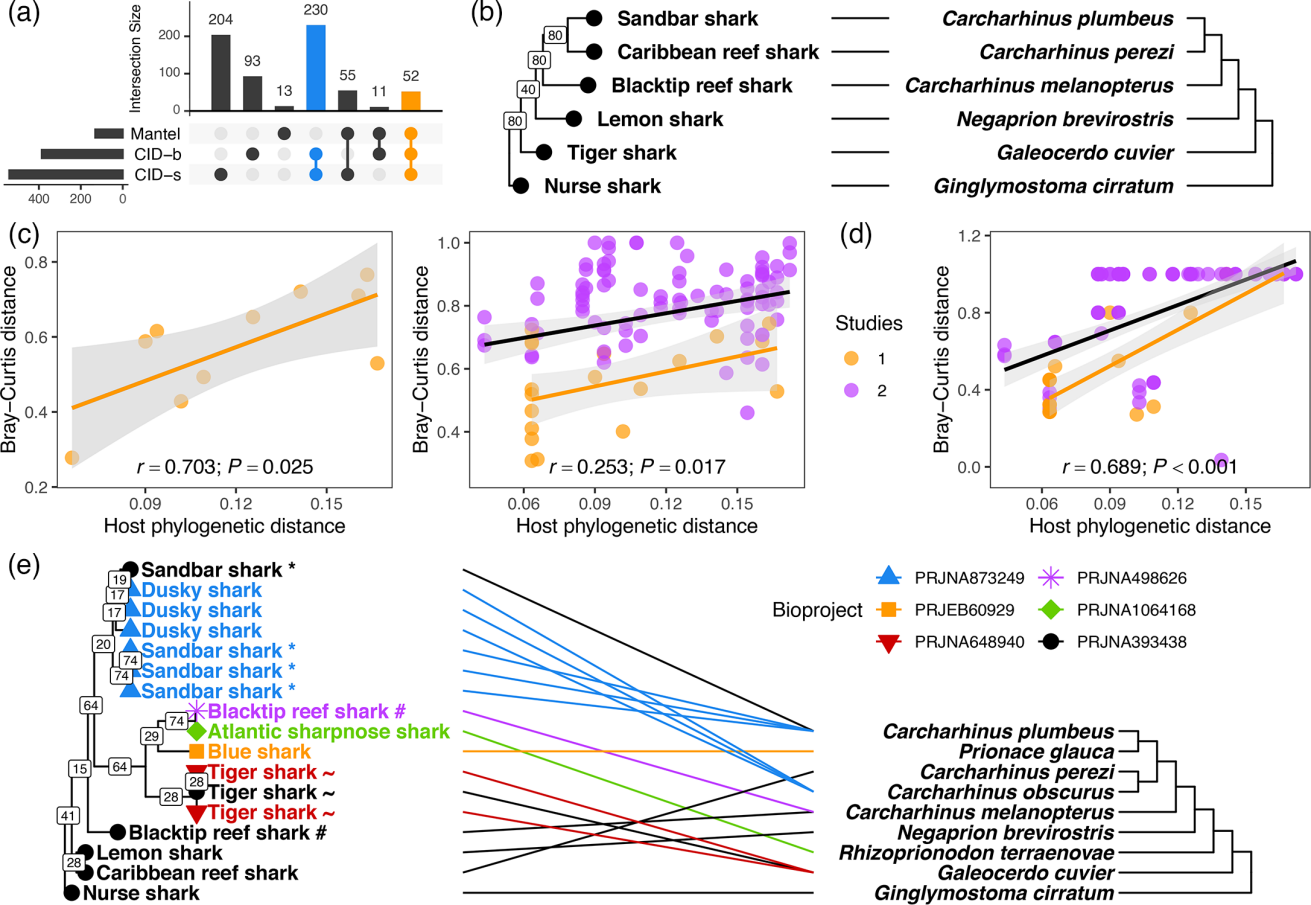
Ephemeral signals of phylosymbiosis in a fraction of the requiem shark dermal microbiome. (**a**) Intersection plot illustrating overlaps between the numbers of OTUs showing evidence of phylosymbiosis among six species from bioproject PRJNA393438 (‘core samples’), using three criteria: perfect topological congruence (CID=0.000) with host phylogeny in bootstrap replicates across OTUs (CID-b) or random subsamples of ten OTUs (CID-s), or strong correlation with host phylogenetic distance (Mantel *r*≥0.9) in random subsamples of ten OTUs. Colours highlight important overlaps between methods. (**b**) Tanglegram illustrating congruence between microbiome structure (Bray–Curtis dissimilarity; left) and host phylogenetic relationships (right), based on 282 out of 844 total OTUs [blue+orange intersections in panel (a)]. Lines indicate perfect correspondence between species. The host cladogram was reproduced from [37], and the dendrograms were rerooted at the nurse shark outgroup. (**c**) Relationships between Bray–Curtis distance and host phylogenetic distance among core samples (left) or all 17 shark samples (right), without the nurse shark outgroup, based on 52 consensus OTUs [orange intersection in panel (a)]. Colours indicate whether each distance was derived from samples within the same study or across two studies. Linear models are fitted (black: all data; yellow: within-study data only) and partial Mantel correlation coefficients (*r*) and *P*-values (1,000 permutations) for the overall relationships (conditioning for across-study comparisons where applicable) are given at the bottom of the panels. (**d**) Partial Mantel correlation plot based on 10 OTUs showing moderately strong evidence of phylosymbiosis among all 17 shark samples. (**e**) Tanglegram among all 17 shark samples based on the same 10 OTUs. Tip symbols and colours indicate independent studies (Table S1). Species replicates across studies are indicated with special characters (* sandbar shark; ~ tiger shark; # blacktip reef shark). Branch labels on dendrograms indicate support values from 1,000 bootstrap replicates across OTUs. Topological congruence is moderate but significant (CID=0.619; *P*<0.001).

Indeed, 174 out of 10,000 bootstrap replicates (1.7 %) of the 6 core samples were perfectly congruent with the host phylogeny (CID=0.000, *P*<0.001). These replicates contained 386 out of 787 OTUs (49.0 %), of which 282 (73.1%) were also identified with a random OTU subsampling procedure (Fig. 3a). However, while the topology of the Bray–Curtis dissimilarity dendrogram was generally well-supported (Fig. 3b), the dissimilarities were not correlated with phylogenetic distances (r=−0.129, *P*=0.558; Fig. S5A). Using a minimum Mantel correlation cutoff of 0.9, the random subsampling procedure also identified 131 OTUs that together supported a significant correlation with phylogenetic distance (*r*=0.679, *P*=0.025), but poor topological congruence with host phylogeny (CID=0.492, *P*=0.103; Fig. S5B). The consensus set of 52 OTUs that were identified both with CID and Mantel criteria showed the strongest correlation with phylogenetic distance (*r*=0.703, *P*=0.025; Fig. 3c) but also did not support topological congruence (CID=0.514, *P*=0.165; Fig. S5C).

The phylosymbiosis signal among the core samples based on these 52 consensus OTUs could, to some extent, be recovered among the full set of 17 shark samples, though the signal was much weaker and noisier (*r*=0.253, *P*=0.017; Fig. 3c) since these OTUs retained evidence of study-specific prevalence based on Jaccard distance (PERMANOVA: *F*_5,11_=4.158, *P*<0.001, *R*^2^=0.654). Bootstrapping across all 1,312 OTUs yielded no replicate that showed congruence with host phylogeny (min CID: 0.711; mean: 0.841±0.031 sd) or an extreme partial Mantel correlation coefficient (mean: −0.114±0.066 sd; max 0.120). However, the random subsampling method identified ten OTUs with relatively strong partial Mantel correlation (*r*=0.689, *P*<0.001; Fig. 3d) and a degree of topological similarity to the host phylogeny driven by improved, though incomplete, grouping by species instead of study (CID=0.619, *P*<0.001; Fig. 3e). These ten OTUs were distinct from the nine core OTUs, though five were also identified among the six core samples (Table 1). These five OTUs support only weak phylosymbiosis across all samples (*r*=0.310, *P*=0.002).

**Table 1.** OTUs implicated in phylosymbiosis in requiem shark dermal microbiomes across 17 shark samples from 9 species and 6 studies Taxonomic annotations are presented to genus level (silva nr99 V138.2 database). The first five OTUs were corroborated when analysing only six core species from the same study that minimizes confounding environmental variation (bioproject PRJNA393438).

Phylum	Class	Order	Family	Genus
Bacillota	Bacilli	Lactobacillales	Lactobacillaceae	*Lactiplantibacillus*
Planctomycetota	Planctomycetes	Pirellulales	Pirellulaceae	*Rhodopirellula*
Pseudomonadota	Gammaproteobacteria	Beggiatoales	Leucotrichaceae	*Cocleimonas*
Pseudomonadota	Gammaproteobacteria	Burkholderiales	Alcaligenaceae	*Pusillimonas*
Thermodesulfobacteriota	Desulfobulbia	Desulfobulbales	Desulfurivibrionaceae	*MSBL7*
Ignavibacteriota	Ignavibacteria	Ignavibacteriales	Melioribacteraceae	*IheB3-7*
Pseudomonadota	Alphaproteobacteria	Caulobacterales	Hyphomonadaceae	*Robiginitomaculum*
Pseudomonadota	Gammaproteobacteria	Burkholderiales	Alcaligenaceae	*Alcaligenes*
Pseudomonadota	Gammaproteobacteria	Burkholderiales	Nitrosomonadaceae	*cr616*
Pseudomonadota	Gammaproteobacteria	Enterobacterales	Aeromonadaceae	*Oceanisphaera*

## Discussion

We presented the first study of phylosymbiosis in the dermal microbiome of nine species within the Carcharhinidae family of sharks, using all available 16S rRNA amplicon sequence data from six previous studies. Despite prior evidence of phylosymbiosis across and within several elasmobranch orders [25,31, 33], we found no general pattern of phylosymbiosis in the overall dermal microbiomes of the Carcharhinidae. Instead, the microbiomes were predominantly structured by study with low levels of OTU sharing, which probably reflects a combination of overarching geographical, temporal and possibly methodological variation. Notwithstanding, we identified minority subsets of OTUs that satisfied either topology-based or distance-based criteria for phylosymbiosis, but there was little consistency in these signals between methods and sample sets, suggesting an obscure, fragile or ephemeral nature of phylosymbiosis. Overall, these results provide only tenuous and provisional evidence for phylosymbiosis within the Carcharhinidae but highlight important factors affecting dermal microbiome structure within this highly diverse elasmobranch family.

Together, the six studies represent six sampling years (2016–2021) and three oceans (West Atlantic Ocean, Mediterranean Sea and Indian Ocean). The taxonomic composition of the microbiomes and the structure observed within each study were consistent with the original publications [20,26,29, 31], highlighting temporal [27,28] and geographical [20,27] structure in dermal microbiomes. Temporal structure at the same site in the Eastern Mediterranean Sea across 3 years (2019–2021) has been observed for sandbar and dusky shark [28] and tiger shark in the West Atlantic from 2018 to 2019 [27]. Localized geographical microbiome structure has been identified for Tiger shark in the West Atlantic [27] and black-tip reef sharks in the Indian Ocean [20]. We did identify the expected structure between species at the same site [28], but replicates of the same species from different studies were more similar to environmental microbiomes from their respective sampling sites than they were to each other. This is consistent with previous findings that microbiomes may vary even among individuals at the same site [28] and indicates that the dermal microbiomes probably primarily reflect environmental commensals, even in migratory species [51,52]. This high variability in dermal microbiomes could, in principle, be compensated for by functional redundancy in disparate bacterial communities [42], as has been shown in temporally variable dermal microbiomes of leopard shark (*Triakis semifasciata*) [53]. However, we found a degree of disparity in functional terms among studies and no evidence of phylosymbiosis when using abundances of functional metabolic terms, which suggests that functional redundancy is unlikely to be compensating for disparity of microbiome composition in Carcharhinidae dermal microbiomes.

Previous studies have identified phylosymbiosis in the elasmobranch dermal microbiome across orders or families. One study examined four species from four orders [25]: thresher shark *Alopias vulpinus* (Lamniformes), leopard shark *T. semifasciata* (Carcharhiniformes), whale shark *Rhincodon typus* (Orectolobiformes) and round ray *Urobatis halleri* (Myliobatiformes). Another study focussed on three species from two orders [33]: horn shark *Heterodontus francisci* (Heterodontiformes), leopard shark *T. semifasciata* (Carcharhiniformes) and swell shark *Cephaloscyllium ventriosum* (Carcharhiniformes). A final study examined three ray species (all Myliobatiformes) and one shark [31]: spiny butterfly ray *Gymnura altavela*, bluntnose stingray *Dasyatis say*, Atlantic stingray *Hypanus sabinus* and Atlantic sharpnose shark *R. terraenovae* (Carcharhiniformes). The peculiar denticle anatomy of shark skin has been suggested to be particularly conducive to phylosymbiosis at deep phylogenetic splits [25], but our results provide no evidence for microbiome-wide ‘shallow’ phylosymbiosis within the Carcharhinidae family. This is broadly consistent with other studies on dermal microbiomes of aquatic animals, for example, in marine fish [54], freshwater fish [55] and marine mammals [56], which found phylosymbiosis between families or higher phylogenetic ranks but little or no evidence within families. Similarly, in gut microbiomes, which have a much more obvious functional role than external microbiomes and usually display strong signals of phylosymbiosis [57], signals of phylosymbiosis may be weak or absent at shallower phylogenetic levels, for example, within brachyurian crab families [58], the Gruidae crane family [59] or the *Tamias* chipmunk genus [60]. However, the dermal microbiomes of salamanders show phylosymbiosis at the genus level [61] and, to varying extents, deeper phylogenetic levels [62,63]. Whole-organism microbiomes in invertebrates also often exhibit shallow phylosymbiosis at the genus level, for example, in marine sponges (*Agelas* [7]) or terrestrial insects (*Nasonia* and *Ceratosolen*) [5,6]. Therefore, while our microbiome-wide results are consistent with the broader literature on marine dermal microbiomes, they do not generally rule out the possibility of shallow phylosymbiosis, possibly only within a fraction of the microbiomes that is masked by the overall variation of the whole microbiome.

Indeed, we demonstrated that simple bootstrap or subsampling methods can be useful for partitioning the total microbiome into fractions based on arbitrary criteria, for example, perfect topological congruence or high correlation with host phylogenetic relationships. This represents a potentially powerful complementary approach to *a priori* partitioning of the microbiome based on OTU taxonomy, prevalence or other criteria [64,65]. Using these methods, we identified ten microbial genera whose abundance patterns across all samples were consistent with phylosymbiosis and may therefore be implicated in functional relationships with the host. For example, *Lactiplantibacillus* and *Alcaligenes* are genera of important keystone symbionts in skin microbiomes that have probiotic, antipathogenic, immunostimulant and microbiome-modulating properties [66,67]. Similarly, *Rhodopirellula*, *Cocleimonas*, *Oceanisphaera* and *Robiginitomaculum* are genera associated with biofilms on kelp, shellfish carapace or elasmobranch skin, though their functions are less clear [68,71]. Conversely, *Pusillimonas*, *MSBL7* and *cr616* are sulphur and ammonia metabolizers that may be of environmental origin rather than strictly associated with shark skin [72,74]. While all these genera are intriguing provisional candidates based on commonly applied statistical criteria for phylosymbiosis [1,3], their functional relevance will require careful validation. Further, we observed a disconnect between topological comparison of dendrograms and association tests between phylogenetic distance and microbiome dissimilarity metrics. We found the Mantel test to be superior in identifying candidate OTUs, whereas dendrogram topologies were unstable and sensitive to the OTU sets included. This echoes previous studies that criticized the topology-based approach due to loss of power and fidelity to true microbiome distances [8,62], although perfect topological congruence is a much more intuitive scoring criterion than an arbitrary cutoff of the Mantel correlation statistic. The general utility of these statistical approaches for scoring subsets of microbiomes against expectations from phylosymbiosis is an open question that requires a cautious approach and future validation work. Notwithstanding, our study has opened a new perspective that emphasizes that the microbiome may vary in the extent of phylosymbiosis among particular microbial subcommunities.

In conclusion, our results question the generality of phylosymbiosis in elasmobranchs and contribute to the controversy over the prevalence of phylosymbiosis at shallow phylogenetic divergence. This may suggest that study effort on phylosymbiosis for conserving the elasmobranch holobiont should be targeted at deeper taxonomic levels such as order level or above to align with previous evidence [25,31, 33]. However, a significant limitation of our study was the reliance on data for only nine species from multiple studies with variable extents of species replication and complex variation in geographical and temporal provenance. Additionally, technical variation around sample processing and primer choice may have introduced further bias to OTU representation [75], although our observation that OTU sharing was poor even between three studies that used the same primers suggests that environmental variation is much more important. Due to the confounded nature of these variables, it was not possible to model batch effects directly to disentangle species effects from study effects, though we attempted to address this problem by careful comparison between the full dataset and a reduced dataset with minimal confounding variation [49] and using partial Mantel tests to account for between-study comparisons. Notwithstanding, our study remains exploratory and highlights that these issues will have to be resolved with coordinated large-scale studies with unified methods that aim to maximize species coverage and minimize confounding and domineering environmental effects on microbiome structure, to allow for robust dissection of phylosymbiotic signal across fractions of the microbiome that may reflect contrasting effects of functional symbionts versus stochastic commensals.

## Supplementary material

10.1099/acmi.0.001140.v3 Uncited Supplementary Material 1. Figures S1-S5

10.1099/acmi.0.001140.v3 Uncited Supplementary Material 2. Tables S1-S3

## Abbreviations

ASV: amplicon sequence variant
CID: clustering information distance
OTU: operational taxonomic unit
PERMANOVA: permutational multivariate analysis of variance
