# Supplementary material for: Ephemeral signatures of phylosymbiosis in dermal microbiomes within the requiem shark family (Carcharhinidae)
Source: Access Microbiol. 2026 Mar 19;8(3):001140.v3. doi: 10.1099/acmi.0.001140.v3 (PMC13022323; doi:10.1099/acmi.0.001140.v3)
Supplement: Uncited Supplementary Material 1. [file acmi-8-01140-s001.pdf]

Ephemeral signatures of phylosymbiosis in dermal microbiomes within the requiem shark family (Carcharhinidae)

Ifan Dafydd Lynn, Marius Alexander Wenzel

March 11, 2026

School of Biological Sciences, University of Aberdeen, Tillydrone Avenue, AB24 2TZ, United Kingdom

Supplemental figures

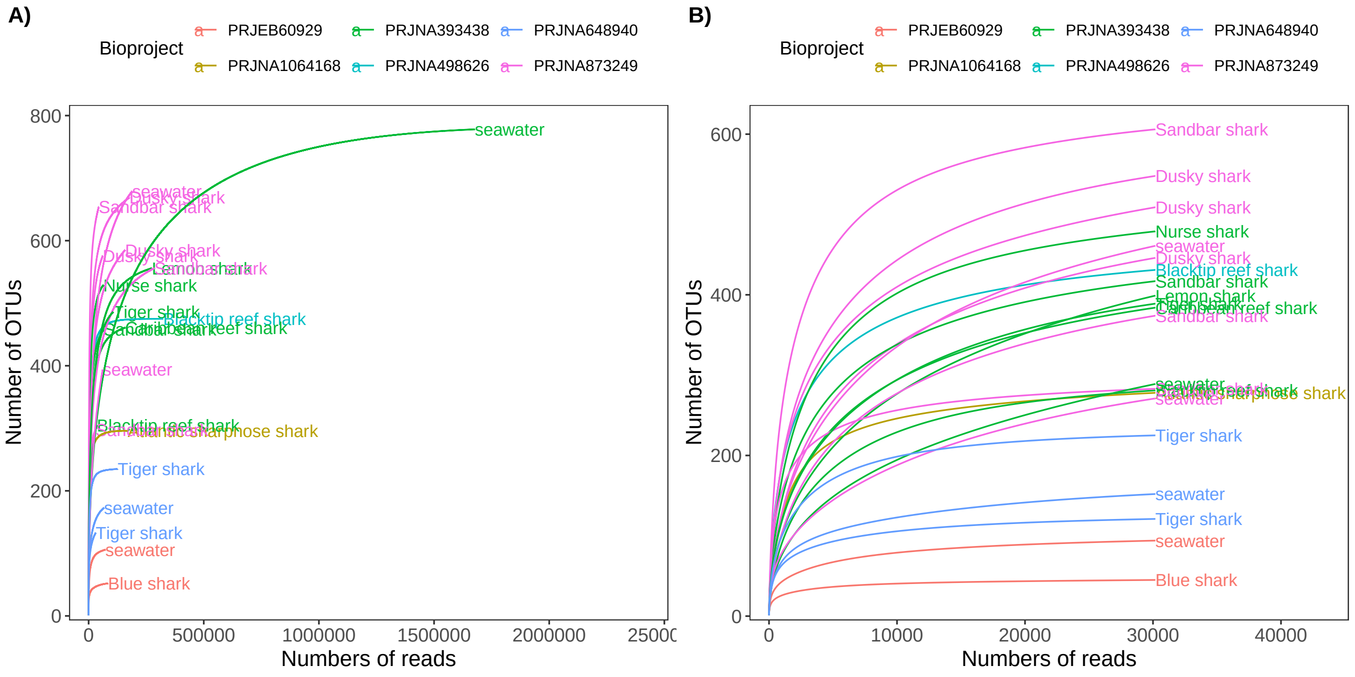

Figure S1: Rarefaction curves based on total read depth of each sample (A) and after rarefaction to the lowest observed read depth (B). Samples are coloured by study (bioproject). Subsampling was carried out using *ggrare* from the *ranacapa* package [1] with step size 10.

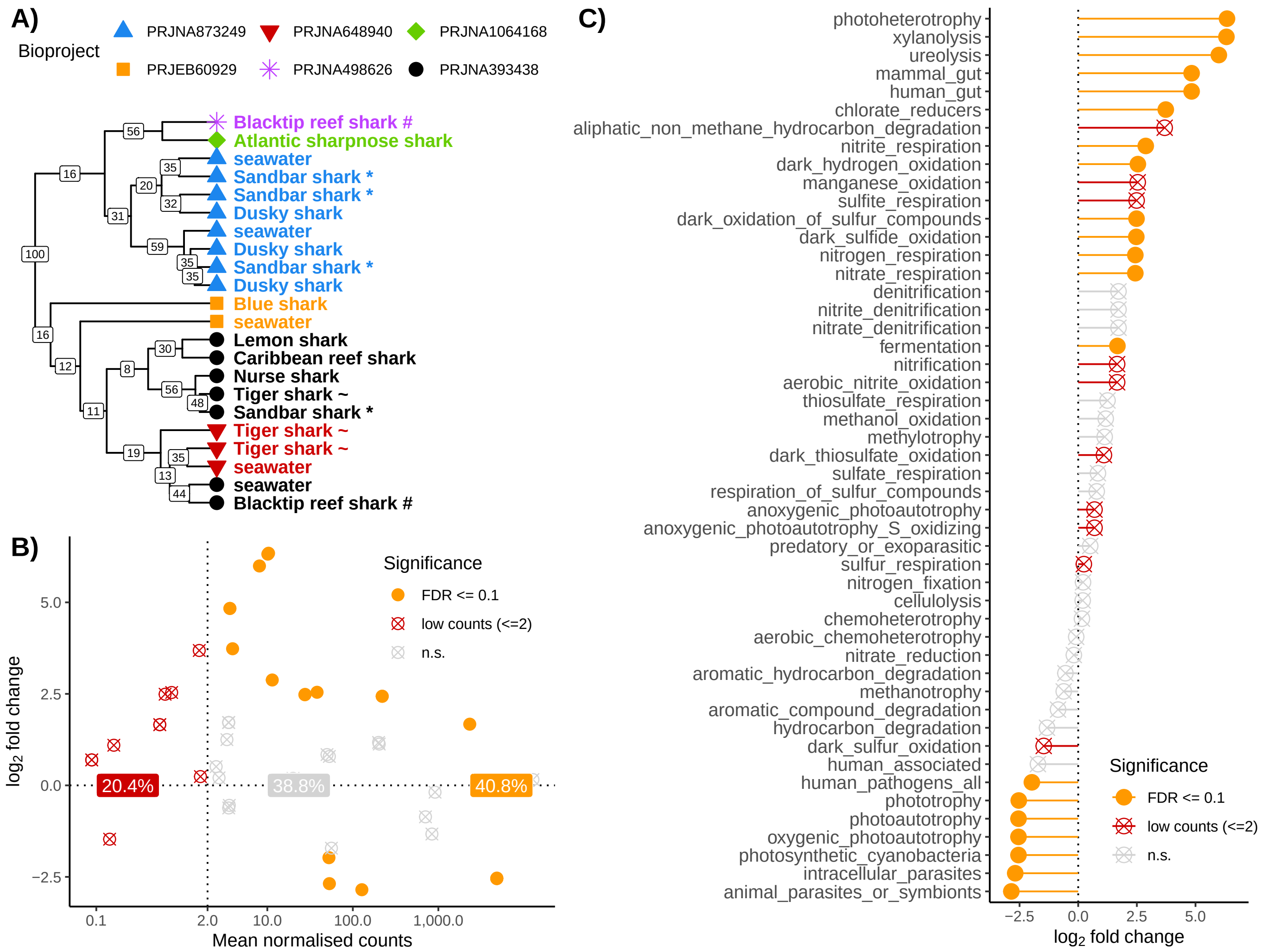

Figure S2: Microbiome structure based on 49 functional terms (FAPROTAX database) assigned to 1328 OTUs from all shark samples and environmental seawater samples. A) Jaccard distance among samples based on sharing of the 49 terms. Tip symbols and colours indicate independent studies (Supplemental Table S1). Species replicates across studies are indicated with special characters (* Sandbar shark; ~ Tiger shark; # Blacktip reef shark). Branch labels indicate support values from 1000 bootstrap replicates across terms. B) Differential abundance of all terms between core samples (bioproject PRJNA393438) and all other samples (others-vs-core). For each term, fold change is plotted against mean normalised abundance, and statistical significance is indicated and tallied with symbols and colours. C) Descriptions of all functional terms, ordered by fold change (others-vs-core). Symbols indicate statistical significance as in panel B.

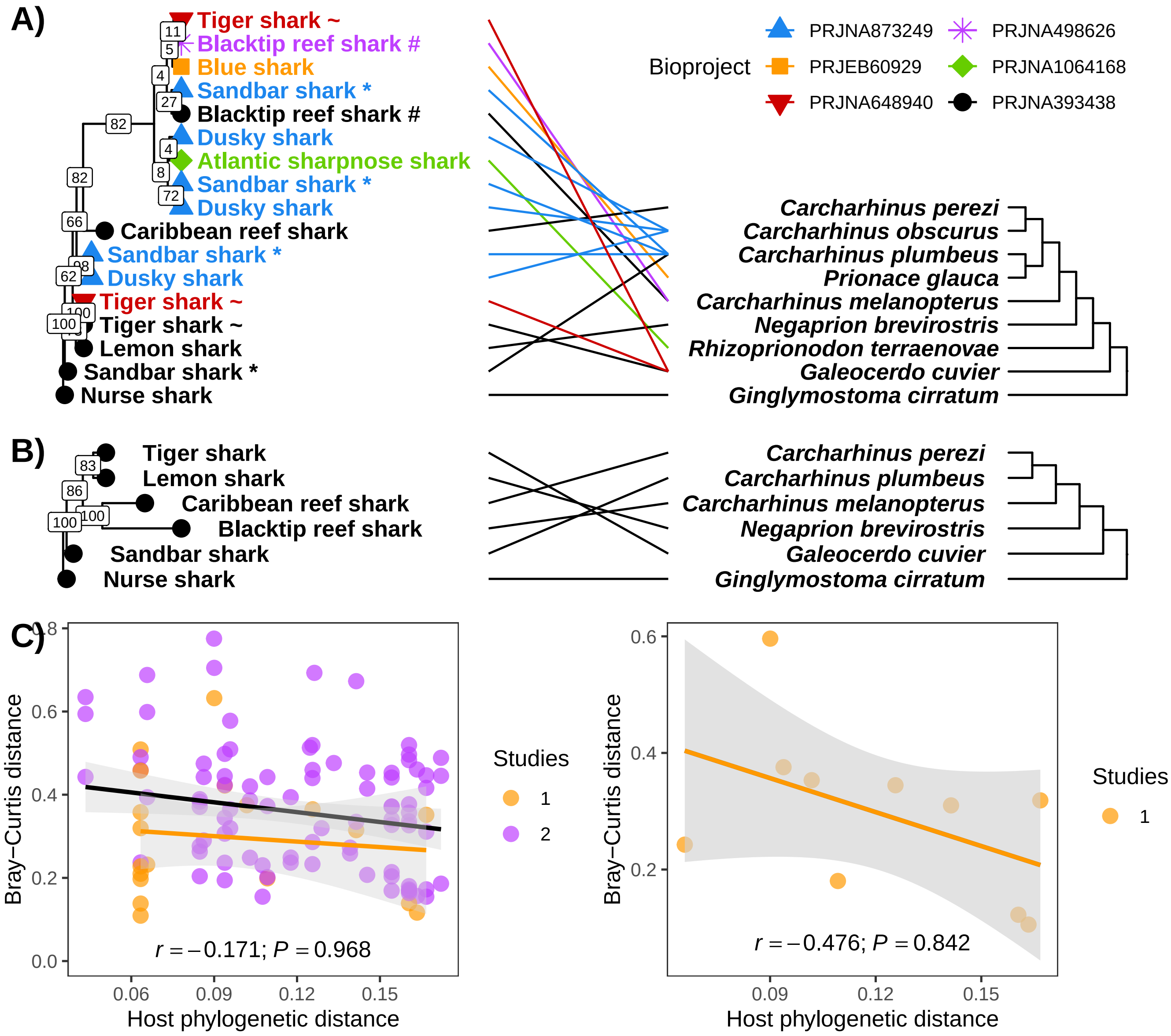

Figure S3: Tanglegrams illustrating lack of correspondence between functional microbiome structure (Bray-Curtis dissimilarity; left) and host phylogenetic relationships (right) for 51 functional terms among all 17 shark samples (A) or 38 terms among six core species from a single study (bioproject PRJNA393438) (B). Tip symbols and colours indicate independent studies (Supplemental Table S1). Species replicates across studies are indicated with special characters (* Sandbar shark; ~ Tiger shark; # Blacktip reef shark). Branch labels on dendrograms indicate support values from 1000 bootstrap replicates across OTUs. The host cladogram was reproduced from [2] and the dendrograms were rerooted at the Nurse shark outgroup. Lines indicate correspondence between species. Both sets of trees are poorly congruent (clustering information distance $CID = 0.810$, $P = 0.294$ and $CID = 0.730$, $P = 0.356$, respectively). (C) Relationships between Bray-Curtis distance and host phylogenetic distance among all samples (left) or core samples (right), without the Nurse shark outgroup. Colours indicate whether distances were derived from samples within the same study or across two studies. Linear models are fitted (black: all data; yellow: within-study data only) and partial Mantel correlation coefficients (r) and P-values (1000 permutations) for the overall relationships (conditioning for across-study comparisons where applicable) are given at the bottom of the panels.

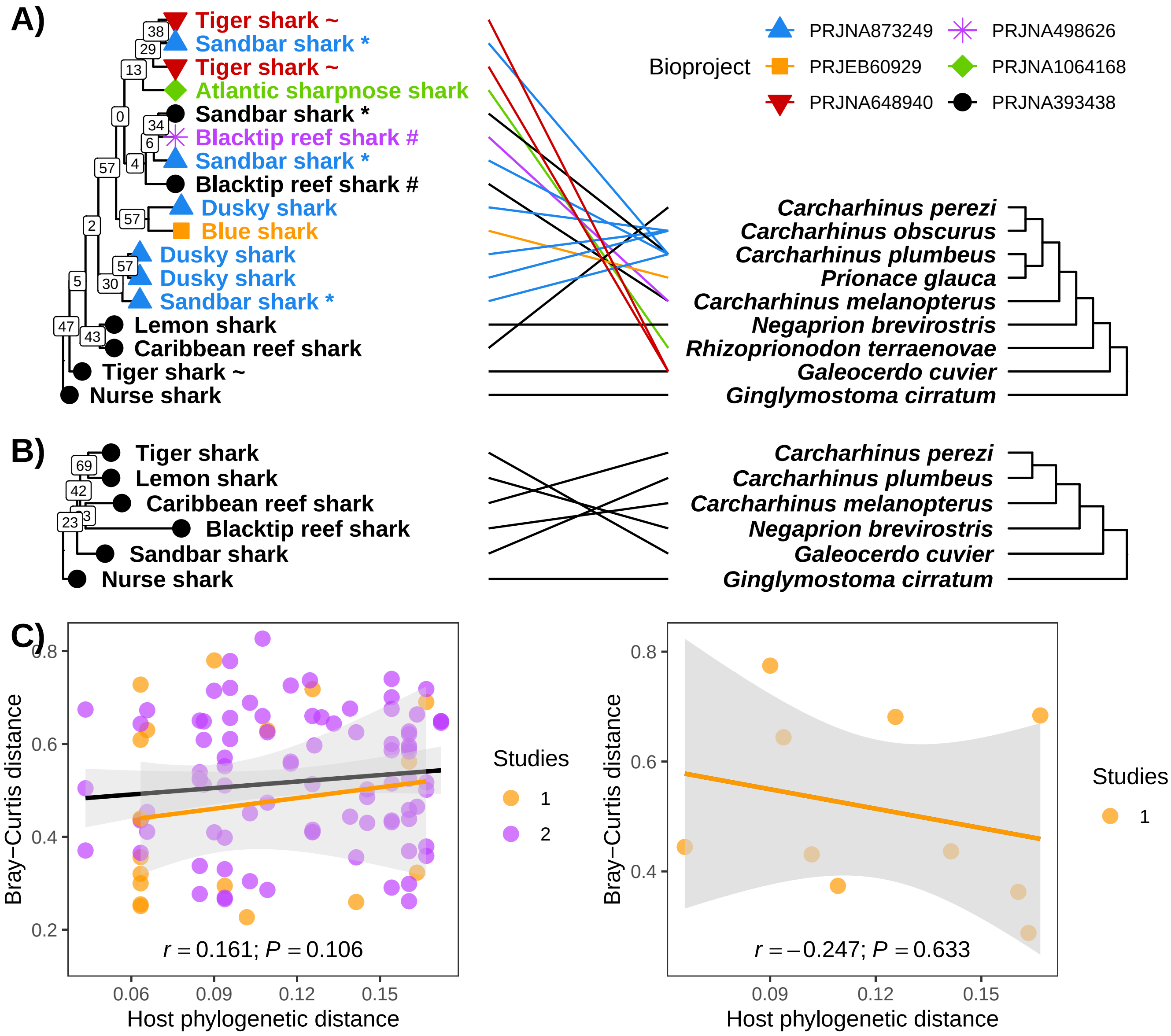

Figure S4: Tanglegrams illustrating lack of correspondence between overall dermal microbiome structure (Bray-Curtis dissimilarity; left) and host phylogenetic relationships (right) for 9 core OTUs present in all samples (A) or 142 core OTUs present in six core species from a single study (bioproject PRJNA393438) (B). Tip symbols and colours indicate independent studies (Supplemental Table S1). Species replicates across studies are indicated with special characters (* Sandbar shark; ~ Tiger shark; # Blacktip reef shark). Branch labels on dendrograms indicate support values from 1000 bootstrap replicates across OTUs. The host cladogram was reproduced from [2] and the dendrograms were rerooted at the Nurse shark outgroup. Lines indicate correspondence between species. Both sets of trees are poorly congruent (clustering information distance $CID = 0.752$, $P = 0.044$ and $CID = 0.730$, $P = 0.364$, respectively). (C) Relationships between Bray-Curtis distance and host phylogenetic distance among all samples (left) or core samples (right), without the Nurse shark outgroup. Colours indicate whether distances were derived from samples within the same study or across two studies. Linear models are fitted (black: all data; yellow: within-study data only) and partial Mantel correlation coefficients (r) and P-values (1000 permutations) for the overall relationships (conditioning for across-study comparisons where applicable) are given at the bottom of the panels.

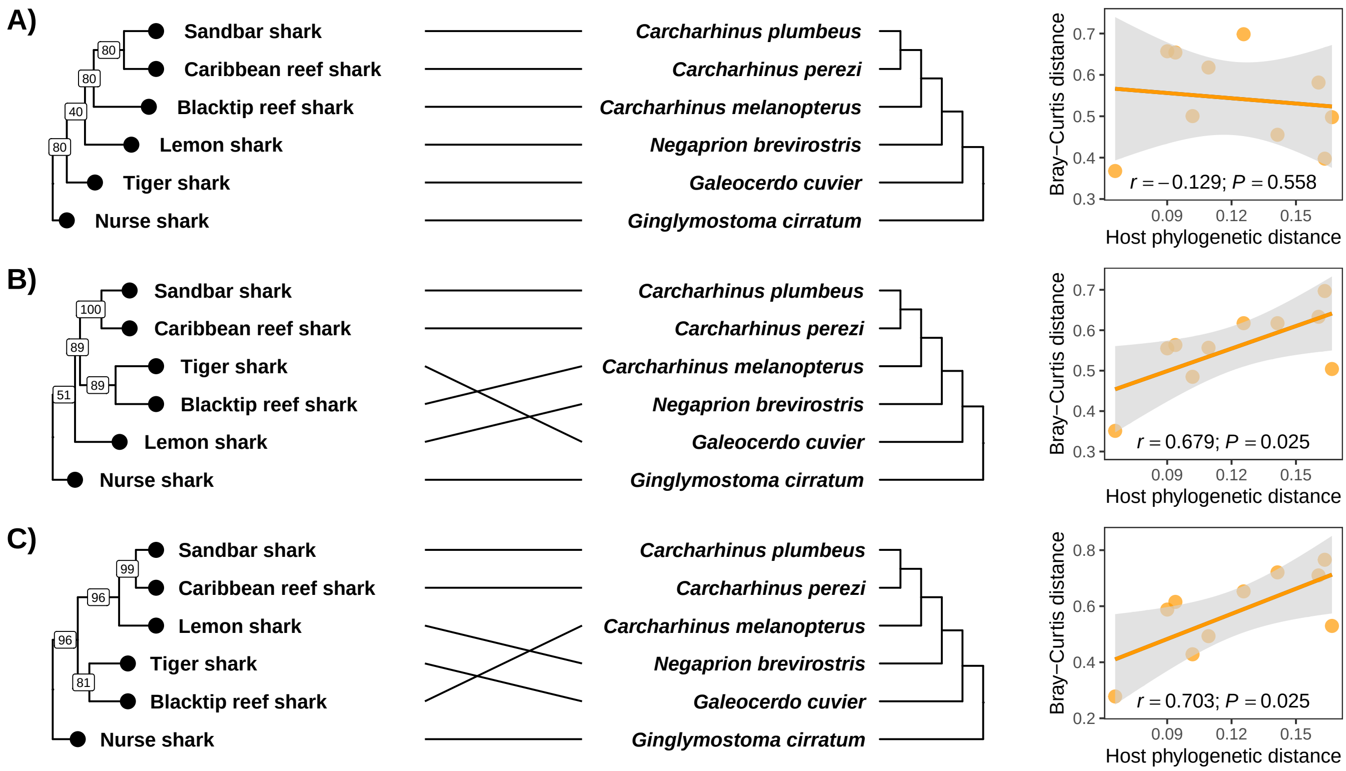

Figure S5: Phylosymbiosis among six core shark species from a single study (bioproject PRJNA393438), based on fractions of the total microbiome identified via bootstrapping or random subsampling across OTUs (10 OTUs at a time). Tanglegrams (left) illustrate topological congruence between hierarchical clustering (Ward's method) of Bray-Curtis microbiome dissimilarity and phylogenetic relationships. Branch labels on dendrograms indicate support values from 1000 bootstrap replicates across OTUs. The host cladogram was reproduced from [2] and the dendrograms were rerooted at the Nurse shark outgroup. Lines indicate correspondence between species. Mantel correlation plots (right) illustrate relationships between Bray-Curtis dissimilarities and phylogenetic distances among five ingroup species (without the Nurse shark outgroup). Linear models are fitted and Mantel correlation coefficients (r) and P-values (1000 permutations) for the relationships are given at the bottom of the panels. A) Results based on 282 OTUs identified based on perfect topological congruence ($CID = 0.000$, $P < 0.001$) alone. B) Results based on 131 OTUs identified based on strong Mantel correlation coefficients ($r \geq 0.9$) alone. C) Results based on 52 OTUs identified based on perfect topological congruence and strong Mantel correlation coefficients.

References

[1] Gaurav S Kandlikar, Zachary J Gold, Madeline C Cowen, Rachel S Meyer, Amanda C Freise, Nathan JB Kraft, Jordan Moberg-Parker, Joshua Sprague, David J Kushner, and Emily E Curd. ranacapa: An r package and shiny web app to explore environmental dna data with exploratory statistics and interactive visualizations. *F1000Research*, 7:1734, 2018.

[2] Baptiste Brée, Fabien L Condamine, and Guillaume Guinot. Combining palaeontological and neontological data shows a delayed diversification burst of carcharhiniform sharks likely mediated by environmental change. *Scientific Reports*, 12(1):21906, 2022.
